# Supplementary material for: Deficiency of the HGF/Met pathway leads to thyroid dysgenesis by impeding late thyroid expansion
Source: Nat Commun. 2024 Apr 11;15:3165. doi: 10.1038/s41467-024-47363-9 (PMC11009301; doi:10.1038/s41467-024-47363-9)
Supplement: Supplementary file 1 — Supplementary Information [file 41467_2024_47363_MOESM1_ESM.pdf]

Supplementary Figures and legends

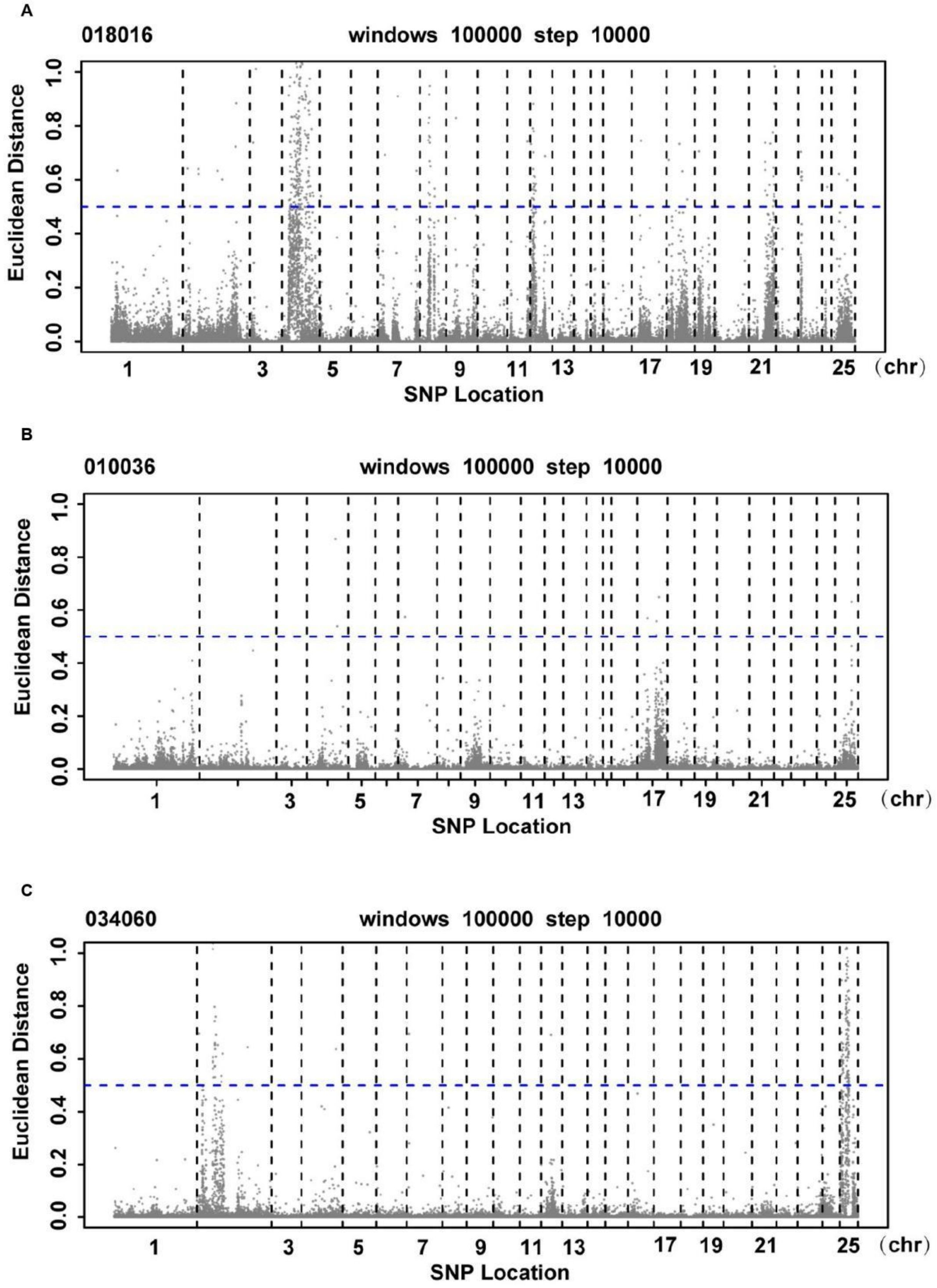

Supplementary Figure 1. Cloning the pathogenic genes from three zebrafish lines with abnormal thyroid morphology by the bulk segregant analysis (BSA). Euclidean distance scores across the genome in the 018016 (A), 010036 (B), and

034060 (C) zebrafish line, respectively. For all panels, vertical dash lines delineated chromosome edges, and chromosome widths represented the relative number of SNPs on the chromosome. Windows:100,000bp. Steps:10,000bp.

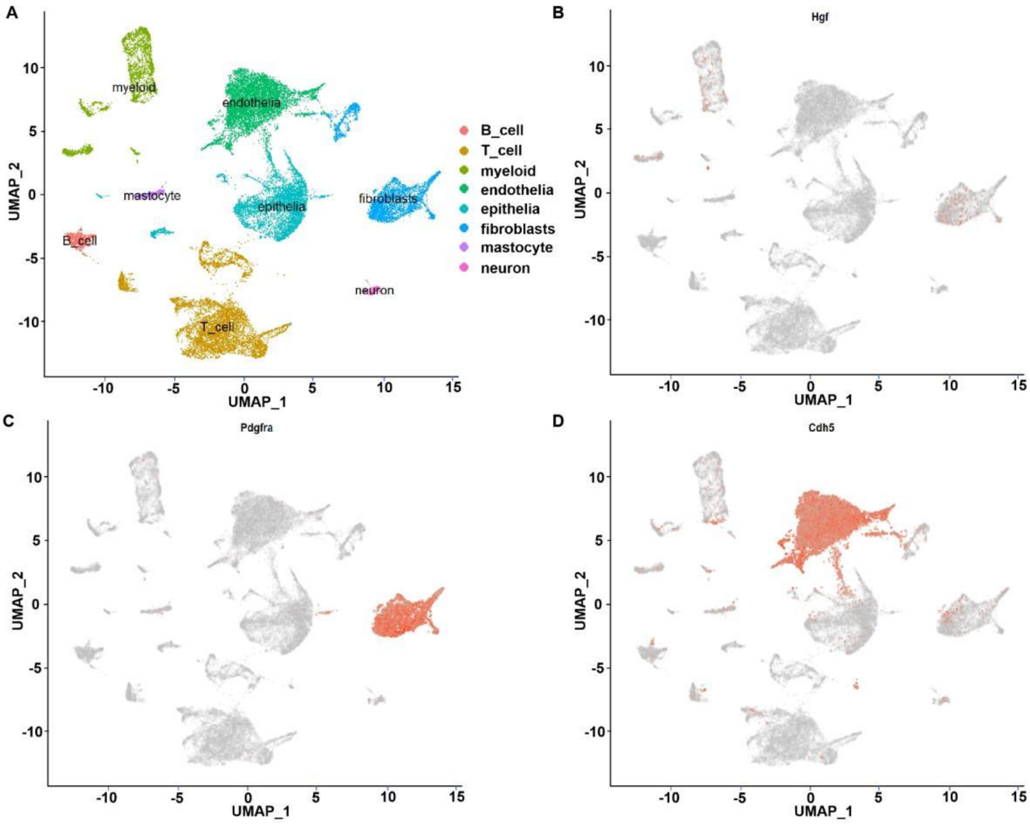

Supplementary Figure 2. Mouse thyroid gland single-cell atlas showing that *Hgf* was mainly expressed in fibroblasts. (A-D) Pdgfra was a specific marker for fibroblasts, Cdh5 was a specific marker for endothelia cells. Hgf was mainly expressed in the fibroblasts, followed by myeloid cells. There was almost no *Hgf* expression of in endothelia cells.

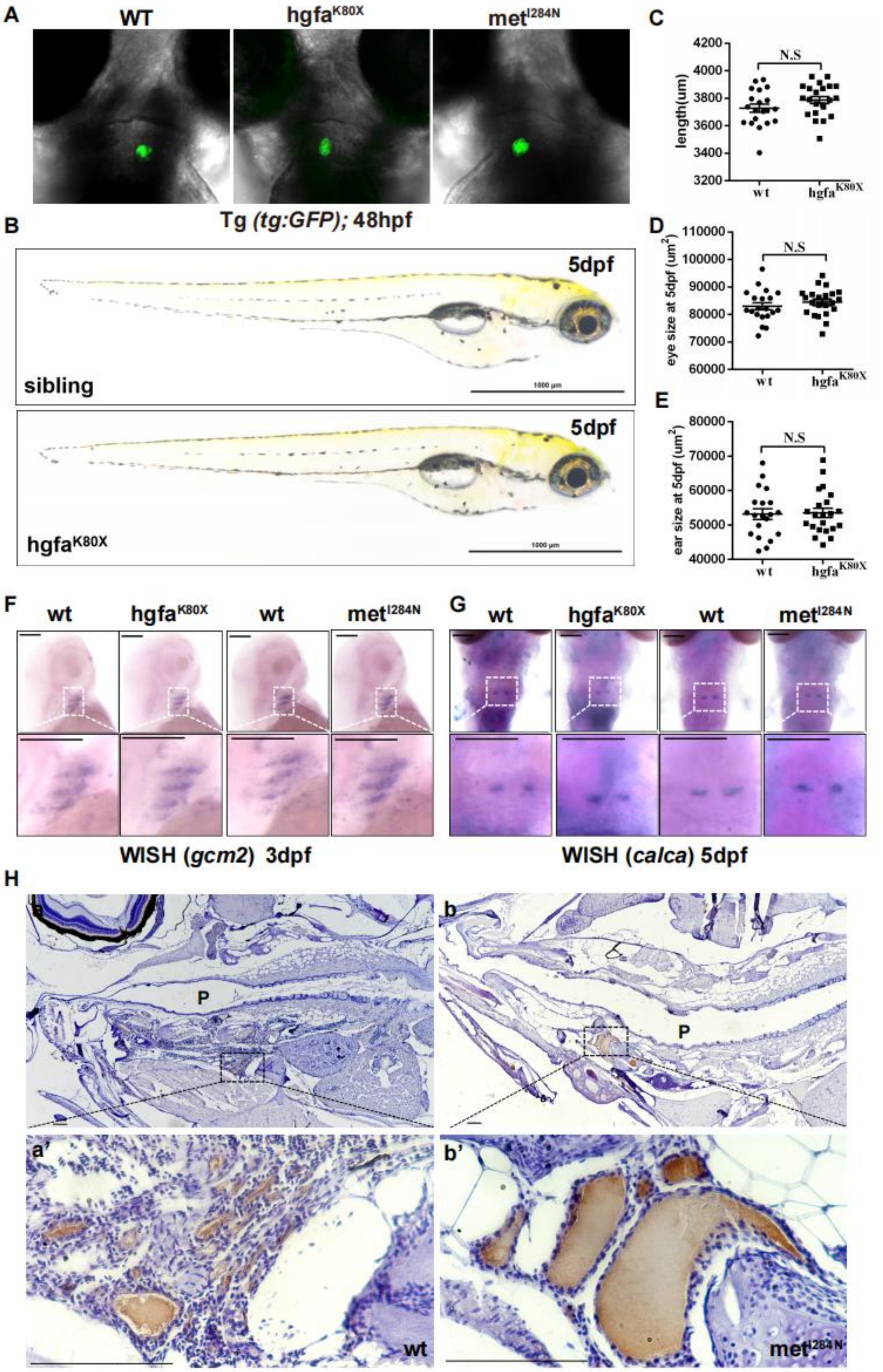

Supplementary Figure 3. Mutant pharyngeal phenotype caused by hgfa/met is restricted to thyroid. (A) Hgfa/met mutants presented normal thyroid primordium at 48 hpf embryos. Tg*(tg:GFP)* transgenic zebrafish lines used for analyzing the

morphology of thyroid primordium at 48 hpf and there was no difference between wildtype and hgfa/met mutant embryos. (B-E) The overall larvae size was unaffected in mutants at 5 dpf, including length (C), eye size (D), and ear size (E). Data are shown as mean ± SEM, n=20 for WT, n=22 for mutants, statistical significance was determined by two sided Student's t test. (F-G) Ultimobranchial gland (marked by *calca* using WISH) and parathyroid (marked by *gcm2* using WISH), another two pharyngeal endoderm derivatives, were unaffected in 3 dpf and 5 dpf mutants, respectively. Scale bars: 100 μm. (H) Immunohistochemical staining with thyroxine of sagittal sections of thyroid follicles in 1.5-month-old WT and $hgfa^{K80X}$ mutated zebrafish (anterior is to the left). Scale bars: 100 μm. Source data are provided as a Source data file.

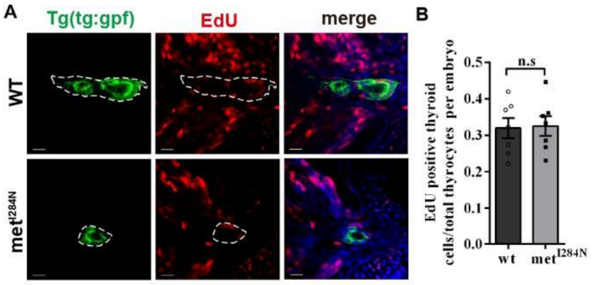

Supplementary Figure 4. The effect of hgf/met deficiency on thyrocytes proliferation in zebrafish larvae at 4 dpf. (A-B) Thyrocytes proliferation in WT and met^{I284N} Tg*(tg:gfp)* transgenic zebrafish embryos, detected by EdU staining. Bars, 50 μm. n.s, not significant. n=7 for each group, anterior is to the right. Data are presented as the mean ± SEM. Group comparisons were performed with two-sided Student's t test. Source data are provided as a Source data file.

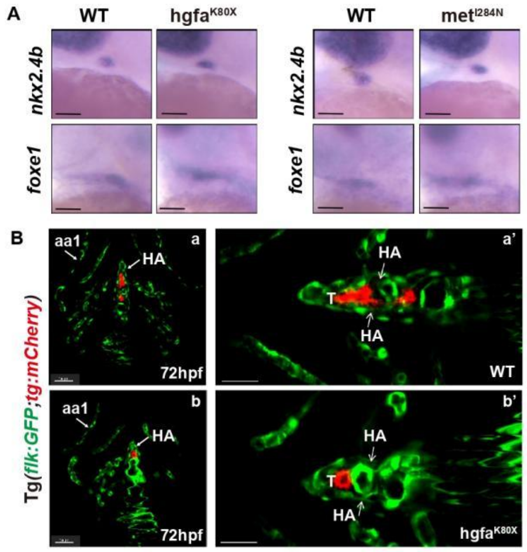

Supplementary Figure 5. The deficiency of *hgfa* or *met* genes does not influence the expression of thyroid transcription factors in thyroid primordium and pharyngeal vessel development. (A) The expression of *nkx2.4b* and *foxe1*, two key transcription factors involved in early thyroid development, did not showed significant difference between the wildtype or homozygous mutant zebrafish embryos at 2 dpf detected by WISH. (B) Tg*(flk1:EGFP;tg:mCherry)* double transgenic lines used for analyzing the effect of deficiency in hgfa on thyroid and surrounding vessels development. As detected, truncated hgfa mutation in zebrafish does not affect the development of vessels surrounding the thyroid at 72 hpf embryos. aa1: aortic arch arteries 1; HA: hypobranchial artery; T: thyroid. Scale bars: 50 μm.

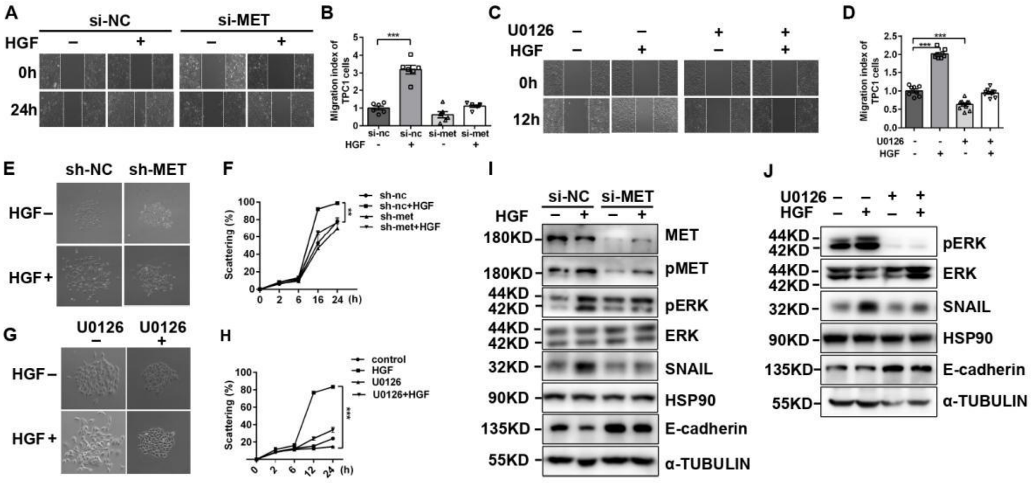

Supplementary Figure 6. HGF/MET downregulates E-cadherin by activating MAPK-snail *in vitro*. (A-D) HGF-induced cell migrating in TPC1 cells can be blocked by *MET* knockdown or treatment with U0126. B, n=6 for each group; D, n=8 for group control and HGF, n=10 for group U0126 and U0126+HGF. (E-H) In TPC1 cells, HGF-induced cell scattering was blocked by *MET* knockdown and U0126 treatment. N=3 for each group. (I, J) By western blot, the effects of HGF-induced increasing expression of Snail and decreased expression of E-cadherin (Cdh1) in TPC1 cells were restored by *MET* knockdown (I) and MEK inhibitors (U0126) (J). Snail, a well-known E-cadherin transcriptional repressor, is activated by MAPK signaling pathway. **represents P<0.01, ***represents P<0.001. Data are presented as the mean ± SEM. Group comparisons were performed with two-sided Student's t test. Source data are provided as a Source data file.

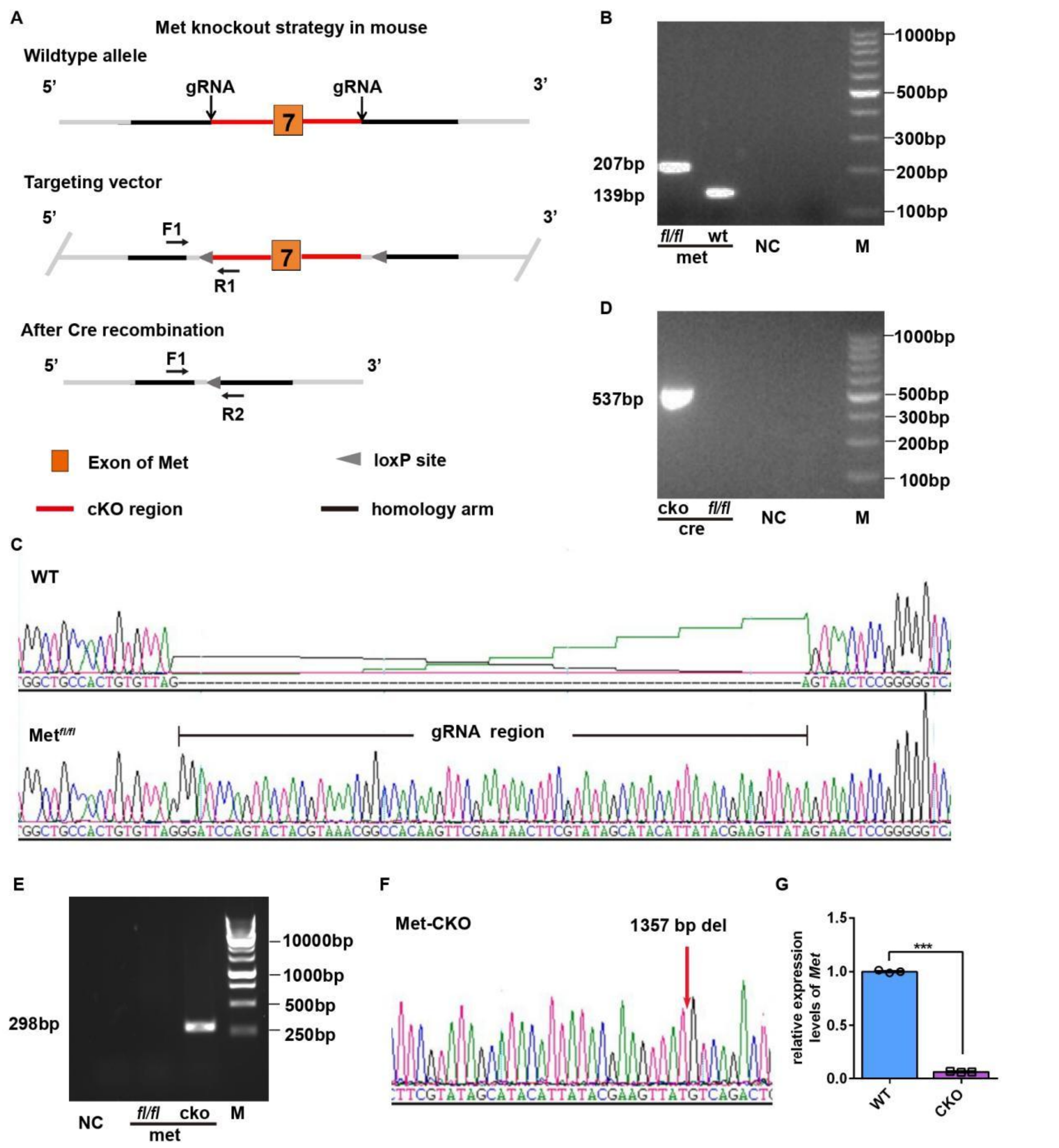

Supplementary Figure 7. Generating a thyroid-specific Met knockout mouse model. (A) Knocking out strategy of mouse *Met*. A floxed Met targeting vector was constructed so that an upstream *loxP* site in the intron preceded exon 7 and the other *loxP* site in the intron immediately downstream of exon 7. The *Met*$^{fl/fl}$ mice were crossed with Pax8-Cre transgenic mice to generate thyroid-specific Met knockout mice. (B-C) Routine genotyping of embryos was performed by PCR with tail DNA

using primers F1/R1. The expected sizes of the loxp allele and wild-type allele were 207 bp and 139 bp, respectively (B). Verification by Sanger sequencing (C). (D) To determine Cre expression, Cre (F/R) primers were used, generating a 537 bp fragment. (E) The thyroid-specific Met deletion were confirmed by primers F1/R2, generating a 298 bp fragment. (F) The size of effective cKO region is 1357 bp, verified by Sanger sequencing. (G) The recombination efficiency was determined by qPCR. n=3, ***represents $P<0.001$. Data are presented as the mean ± SEM. Group comparisons were performed with two-sided Student's t test. Source data are provided as a Source data file.

Supplementary Table 1. Fine genetic mapping of 018016 family

chromsome	postion	reference	alteration	mut_vaf	wt_vaf	variation	gene name	mutation	case-ref	wt-alt
chr4	10650696	C	A	0.884146	0.487395	MISSENSE	NET1	c.145G>T; p.ASP49TYR		
chr4	11334461	G	A	0.876289	0.467647	MISSENSE	CNOT4_(2_OF_2)	c.764C>T; p.SER255LEU		
chr4	15177339	A	C	0.874016	0.42268	MISSENSE	ATP2B1A	c.57T>G; p.ASN19LYS	2/60	0/16
chr4	18670375	T	A	0.989744	0.283871	STOP_GAINED	HGFA	c.238A>T; p.LYS80*	0/100	0/16
chr4	31962425	C	A	0.739496	0.286822	MISSENSE	BX324003.3	c.759C>A; p.ASP253GLU	16/16	0/16
chr4	36797341	C	G	0.804233	0.266667	MISSENSE	SI:CH211-209N20.1	c.956C>G; p.THR319ARG		
chr4	36797355	G	C	0.796512	0.2	MISSENSE	SI:CH211-209N20.1	c.970G>C; p.GLU324GLN		
chr4	36797368	G	A	0.809524	0.166667	MISSENSE	SI:CH211-209N20.1	c.983G>A; p.ARG328LYS		
chr4	41208041	G	A	0.884876	0.0625	MISSENSE	SI:DKEY-238G7.1	c.301G>A; p.ALA101THR		
chr4	41208151	A	T	0.97551	0.176471	MISSENSE	SI:DKEY-238G7.1	c.411A>T; p.GLN137HIS		
chr4	42245177	A	G	0.708333	0.375	MISSENSE	SI:DKEY-250K10.1	c.2110T>C; p.PHE704LEU		
chr4	42383693	T	G	0.977273	0.314741	MISSENSE	SI:DKEY-8O9.2	c.2488A>C; p.MET830LEU		
chr4	42383713	T	G	0.972763	0.321429	MISSENSE	SI:DKEY-8O9.2	c.2468A>C; p.GLN823PRO		
chr4	42486422	T	A	0.96338	0.352668	MISSENSE	BX571954.1	c.1409T>A; p.VAL470ASP		
chr4	48351435	C	T	0.723333	0.27907	MISSENSE	SI:DKEY-9I5.4	c.874G>A; p.GLY292ARG		
chr4	51655360	C	T	0.741573	0.171053	MISSENSE	SI:DKEYP-80D11.10	c.3253C>T; p.ARG1085CYS		
chr4	52293887	C	A	0.833333	0.130435	MISSENSE	SI:CH211-42I6.2	c.235C>A; p.GLN79LYS		
chr4	55460525	A	G	0.894309	0.304878	MISSENSE	SI:DKEY-4E4.1	c.649A>G; p.THR217ALA		
chr4	57103814	G	C	0.857143	0.085561	MISSENSE	SI:CH211-207E19.2	c.2881C>G; p.HIS961ASP		
chr4	57103824	G	C	0.892857	0.102703	MISSENSE	SI:CH211-207E19.2	c.2871C>G; p.HIS957GLN		
chr4	57103828	C	A	0.903226	0.103825	MISSENSE	SI:CH211-207E19.2	c.2867G>T; p.TRP956LEU		
chr4	57103829	A	T	0.904762	0.104972	MISSENSE	SI:CH211-207E19.2	c.2866T>A; p.TRP956ARG		
chr4	57103896	A	T	0.775194	0.212928	MISSENSE	SI:CH211-207E19.2	c.2799T>A; p.ASP933GLU		
chr4	57103900	C	A	0.773438	0.212121	MISSENSE	SI:CH211-207E19.2	c.2795G>T; p.GLY932VAL		
chr4	57835910	A	G	0.825688	0.160584	MISSENSE	SI:DKEYP-33C10.7	c.335A>G; p.LYS112ARG		

case-ref: indicates that reference nucleotide was verificated in phenotypic embryos by Sanger sequencing;
wt-alt: indicates that alteration nucleotide was verificated in wild type zebrafish by Sanger sequencing.

Supplementary Table 2. Fine genetic mapping of 010036 family

chromsome	postion	reference	alteration	mut_vaf	wt_vaf	variation	gene name	mutation
chr1	37651562	T	A	1	0.375	MISSENSE	SI:CH211-76M11.5	c.2862T>A; p.PHE954LEU
chr2	11579679	T	C	0.761905	0.477612	MISSENSE	GREB1L	c.1397T>C; p.LEU466PRO
chr2	47444498	C	T	0.741935	0	MISSENSE	CABZ01078499.2	c.2836C>T; p.PRO946SER
chr4	43456926	A	T	1	0	MISSENSE	SI:DKEY-22A18.2	c.2616A>T; p.LYS872ASN
chr4	51814522	A	*,C	0.872727	0	MISSENSE	SI:CH211-286L10.1	c.1586A>C; p.TYR529SER
chr5	39094997	T	A	0.719101	0.492063	MISSENSE	CU984579.1	c.236T>A; p.MET79LYS
chr5	39095008	A	G	0.722892	0.490909	MISSENSE	CU984579.1	c.247A>G; p.LYS83GLU
chr7	3894900	C	A	0.916667	0.454545	MISSENSE	SI:CH211-282J17.8	c.370G>T; p.VAL124PHE
chr7	4023157	A	T	1	0	MISSENSE	SI:DKEY-88N24.7	c.75T>A; p.ASN25LYS
chr7	4023164	A	G	1	0	MISSENSE	SI:DKEY-88N24.7	c.68T>C; p.MET23THR
chr7	4023167	C	T	1	0	MISSENSE	SI:DKEY-88N24.7	c.65G>A; p.ARG22LYS
chr7	4096558	T	C,*	0.802326	0	MISSENSE	BX284672.1	c.358A>G; p.THR120ALA
chr8	2363858	A	T	0.857143	0	MISSENSE	CT583679.1	c.484A>T; p.THR162SER
chr10	30768244	G	A	0.736842	0.303571	MISSENSE	C10H11ORF63	c.540G>A; p.MET180ILE
chr10	30768248	G	A	0.741379	0.320755	MISSENSE	C10H11ORF63	c.544G>A; p.VAL182ILE
chr10	30768290	T	G	0.712329	0.263158	MISSENSE	C10H11ORF63	c.586T>G; p.SER196ALA
chr17	52246374	G	C	0.820513	0.310345	MISSENSE	NOL10	c.2117G>C; p.GLY706ALA
chr24	21020005	G	C	0.71875	0.44186	MISSENSE	PLCD1A	c.1913G>C; p.ARG638THR
chr25	14078184	A	T	0.964912	0.387755	STOP_GAINE	CK2A2B	c.630T>A; p.TYR210*
chr25	19075276	G	A	0.974684	0.225806	MISSENSE	MET	c.649G>A; p.GLU217LYS
chr25	19422984	C	T	1	0.235294	MISSENSE	NT5DC3	c.1060G>A; p.ALA354THR

Supplementary Table 3. Fine genetic mapping of 034060 family

chromsome	postion	reference	alteration	mut_vaf	wt_vaf	variation	gene name	mutation
chr2	16093087	G	T	0.700422	0	MISSENSE	ARHGEF4	c.1787C>A; p.PRO596GLN
chr4	3657228	G	A	0.708333	0.352941	MISSENSE	CABZ01076533.1	c.1166C>T; p.SER389LEU
chr4	34248591	A	T	0.870968	0	MISSENSE	SI:DKEY-207M2.4	c.1373T>A; p.PHE458TYR
chr4	39227190	G	A	0.863636	0	MISSENSE	TRIM14_(16_OF_17)	c.289G>A; p.GLU97LYS
chr4	39227191	A	G	0.869565	0	MISSENSE	TRIM14_(16_OF_17)	c.290A>G; p.GLU97GLY
chr4	39227196	A	G	0.84	0	MISSENSE	TRIM14_(16_OF_17)	c.295A>G; p.LYS99GLU
chr4	39227199	A	T	0.846154	0	MISSENSE	TRIM14_(16_OF_17)	c.298A>T; p.SER100CYS
chr4	42343152	T	G	1	0	MISSENSE	SI:DKEY-8O9.6	c.231A>C; p.LYS77ASN
chr4	44431365	A	G	1	0	MISSENSE	SI:DKEY-257E4.5	c.1153A>G; p.THR385ALA
chr4	44431386	T	A	1	0	MISSENSE	SI:DKEY-257E4.5	c.1174T>A; p.LEU392MET
chr4	44431387	T	A	1	0	STOP_GAINED	SI:DKEY-257E4.5	c.1175T>A; p.LEU392*
chr4	45905651	A	T	1	0	MISSENSE	CR450785.2	c.1328T>A; p.ILE443ASN
chr4	45905654	C	A	1	0	MISSENSE	CR450785.2	c.1325G>T; p.ARG442MET
chr4	52313101	T	A	0.913043	0	MISSENSE	SI:CH211-42I6.2	c.2214T>A; p.ASN738LYS
chr4	55914869	A	T,*	0.73913	0	MISSENSE	SI:DKEY-196N19.7	c.145T>A; p.SER49THR
chr7	50230038	T	C	0.769231	0.44186	MISSENSE	ACANA	c.1342T>C; p.SER448PRO
chr7	51344775	T	C	0.703704	0.48	MISSENSE	CD44A	c.574A>G; p.THR192ALA
chr8	2364257	G	T	1	0	MISSENSE	CT583679.1	c.883G>T; p.ALA295SER
chr8	2404776	C	T	0.730769	0.477273	MISSENSE	SI:DKEYP-117B11.2	c.1924G>A; p.GLY642SER
chr11	18663825	T	*,C	0.772727	0	MISSENSE	CABZ01112215.2	c.905A>G; p.TYR302CYS
chr11	18663826	A	*,T	0.772727	0	MISSENSE	CABZ01112215.2	c.904T>A; p.TYR302ASN
chr12	47020314	C	A	0.709677	0	MISSENSE	CABZ01048958.1	c.1490G>T; p.TRP497LEU
chr13	7020171	G	A	0.710526	0.489362	MISSENSE	NINL	c.616G>A; p.GLY206ARG
chr17	52246374	G	C	0.72	0.36	MISSENSE	NOL10	c.2117G>C; p.GLY706ALA
chr19	11320174	T	C	0.761905	0.366667	MISSENSE	SLC9A3.1	c.2188A>G; p.ILE730VAL
chr20	42830675	A	C	1	0	MISSENSE	SI:CH211-215M21.11	c.1072A>C; p.THR358PRO
chr20	42869172	A	C	0.77193	0	MISSENSE	SI:CH211-215M21.13	c.407A>C; p.GLN136PRO
chr25	2859707	G	T	0.710145	0	MISSENSE	CABZ01048092.1	c.684C>A; p.PHE228LEU
chr25	19075478	T	A	1	0.425	MISSENSE	MET	c.851T>A; p.ILE284ASN

Supplementary Table 4. Oligonucleotides used in this paper

Gene	Primer ID	Sequence (5' to 3')
tshbα	tshbα probe F	TTAATGAAGGTTGCCGTGCC
tshbα	tshbα probe R	TCCTCGGGGTACAGATGATG
tg	tg probe F	GTACCACTTACCTGAAAACG
tg	tg probe R	TGCTTGGAGTCAGAGTGAAG
gcm2	gcm2 probe F	CTACTACAATAGTGACTACC
gcm2	gcm2 probe R	AATTTTTTATTCCTTTAAAAC
calca	calca probe F	ATGGTTATGTTGAAGATCTCC
calca	calca probe R	AGTGTCCATTCCCAGGGTTC
nis	nis probe F	ATGAGGTTTGGCAGAGGGATG
nis	nis probe R	AACCAGACCATAGTGCCACCC
tpo	tpo probe F	CTTGGACAGA GTTGAATCTC
tpo	tpo probe R	GCATCATCAGGCTGTTCTCT
nkx2.4b	nkx2.4b probe F	ATGTCCTTGAGCCCCAAACA
nkx2.4b	nkx2.4b probe R	TCCATTCCCGGTAACGTGC
foxe1	foxe1 probe F	ATGCCTGTGGTTAAAGTGGA
foxe1	foxe1 probe R	ACAAACCTGTCCATATTACC
met	met probe F	TGTGGTCATCGCAAGAGAAG
met	met probe R	AGTTCCCACAGCAAAACACC
hgfa	hgfa probe F	TGGACTGCCTCAGTGTTCAG
hgfa	hgfa probe R	AATGCTGACCACCCAACTTC
