# Supplementary material for: Deficiency of the HGF/Met pathway leads to thyroid dysgenesis by impeding late thyroid expansion
Source: Nat Commun. 2024 Apr 11;15:3165. doi: 10.1038/s41467-024-47363-9 (PMC11009301; doi:10.1038/s41467-024-47363-9)
Supplement: Supplementary file 3 — Description of Additional Supplementary Files [file 41467_2024_47363_MOESM3_ESM.pdf]

Description of Additional Supplementary Files

Supplementary Movie 1. Time-lapse imaging of wild-type Tg(*tg:GFP*) embryo. GFP labels the thyroid. The live imaging was started at 56 hpf and covered 16 h. Ventral view, anterior to the left.

Supplementary Movie 2. Time-lapse imaging of *hgfa* mutant Tg(*tg:GFP*) embryo. GFP labels the thyroid. The live imaging was started at 56 hpf and covered 16 h. Ventral view, anterior to the left.

Supplementary Movie 3. Time-lapse imaging of met mutant Tg(*tg:GFP*) embryo. GFP labels the thyroid. The live imaging was started at 56 hpf and covered 16 h. Ventral view, anterior to the left.

Supplementary Movie 4. Time-lapse imaging of a U0126-treated Tg(*flk1:GFP;tg:mCherry*) embryo. GFP labels vascular and mCherry labels thyroid. The live imaging was started at 60 hpf and covered 12 h. Ventral view, anterior to the left. Thyroid primordium in the U0126-treated embryos failed to caudally expand along the pharyngeal midline during the late stage of thyroid development from 60 hpf to 72 hpf.
