# Supplementary material for: Deficiency of the HGF/Met pathway leads to thyroid dysgenesis by impeding late thyroid expansion
Source: Nat Commun. 2024 Apr 11;15:3165. doi: 10.1038/s41467-024-47363-9 (PMC11009301; doi:10.1038/s41467-024-47363-9)
Supplement: Supplementary file 8 — Reporting Summary [file 41467_2024_47363_MOESM8_ESM.pdf]

nature portfolio

Corresponding author(s): Huai-dong Song;Mei Dong;Shuang-Xia Zhao

Last updated by author(s): Feb 26, 2024

Reporting Summary

Nature Portfolio wishes to improve the reproducibility of the work that we publish. This form provides structure for consistency and transparency in reporting. For further information on Nature Portfolio policies, see our Editorial Policies and the Editorial Policy Checklist.

Statistics

For all statistical analyses, confirm that the following items are present in the figure legend, table legend, main text, or Methods section.

n/a	Confirmed	
☐	☒	The exact sample size (*n*) for each experimental group/condition, given as a discrete number and unit of measurement
☐	☒	A statement on whether measurements were taken from distinct samples or whether the same sample was measured repeatedly
☐	☒	The statistical test(s) used AND whether they are one- or two-sided<br>*Only common tests should be described solely by name; describe more complex techniques in the Methods section.*
☒	☐	A description of all covariates tested
☒	☐	A description of any assumptions or corrections, such as tests of normality and adjustment for multiple comparisons
☐	☒	A full description of the statistical parameters including central tendency (e.g. means) or other basic estimates (e.g. regression coefficient) AND variation (e.g. standard deviation) or associated estimates of uncertainty (e.g. confidence intervals)
☐	☒	For null hypothesis testing, the test statistic (e.g. *F*, *t*, *r*) with confidence intervals, effect sizes, degrees of freedom and *P* value noted<br>*Give P values as exact values whenever suitable.*
☒	☐	For Bayesian analysis, information on the choice of priors and Markov chain Monte Carlo settings
☒	☐	For hierarchical and complex designs, identification of the appropriate level for tests and full reporting of outcomes
☒	☐	Estimates of effect sizes (e.g. Cohen's *d*, Pearson's *r*), indicating how they were calculated

Our web collection on statistics for biologists contains articles on many of the points above.

Software and code

Policy information about availability of computer code

Data collection	Imaging data was collected using NIS Elements (Nicon, V4.50.00)
Data analysis	Group comparisons of normally distributed data were performed with an unpaired Student’s t test. SPSS 19.0 software (IBM, Chicago, IL, USA) was used for all statistical analyses. Statistical significance was set at P < 0.05. Diagrams were made using GraphPad Prism 6. Imaris 9.0.1 was used for analysis of image data. For single cell RNA sequencing, data was processed using CellRanger (v3.0.1) recommended pipeline. Further analysis was conducted in R (V3.6.1) using Seurat (V3.1.1).

For manuscripts utilizing custom algorithms or software that are central to the research but not yet described in published literature, software must be made available to editors and reviewers. We strongly encourage code deposition in a community repository (e.g. GitHub). See the Nature Portfolio guidelines for submitting code & software for further information.

Data

Policy information about availability of data

All manuscripts must include a data availability statement. This statement should provide the following information, where applicable:

- Accession codes, unique identifiers, or web links for publicly available datasets
- A description of any restrictions on data availability
- For clinical datasets or third party data, please ensure that the statement adheres to our policy

All data associated with this study are presented in the paper and the Supplementary Materials. All software and algorithms used in this study are freely or commercially available and are are listed in the Methods section. The resulting fastq files were aligned to the mouse reference genome (mm10) (https://

www.ncbi.nlm.nih.gov/datasets/genome/GCF_000001635.20/). Source data are provided with this paper.

Research involving human participants, their data, or biological material

Policy information about studies with human participants or human data. See also policy information about sex, gender (identity/presentation), and sexual orientation and race, ethnicity and racism.

Reporting on sex and gender	N/A
Reporting on race, ethnicity, or other socially relevant groupings	N/A
Population characteristics	N/A
Recruitment	N/A
Ethics oversight	N/A

Note that full information on the approval of the study protocol must also be provided in the manuscript.

Field-specific reporting

Please select the one below that is the best fit for your research. If you are not sure, read the appropriate sections before making your selection.

[x] Life sciences [ ] Behavioural & social sciences [ ] Ecological, evolutionary & environmental sciences

For a reference copy of the document with all sections, see nature.com/documents/nr-reporting-summary-flat.pdf

Life sciences study design

All studies must disclose on these points even when the disclosure is negative.

Sample size	No statistical method was used to predetermine sample size. Sample sizes were chosen based on sample availability and fundation.
Data exclusions	No data were excluded for analysis.
Replication	For each representative image/data, experiments were performed at least three times at the same condition with consistent results .
Randomization	All samples were randomly allocated into groups.
Blinding	Blinding was not possible as groups were divided by different treatments.

Reporting for specific materials, systems and methods

We require information from authors about some types of materials, experimental systems and methods used in many studies. Here, indicate whether each material, system or method listed is relevant to your study. If you are not sure if a list item applies to your research, read the appropriate section before selecting a response.

Materials & experimental systems

n/a	Involved in the study	
[ ]	[x]	Antibodies
[ ]	[x]	Eukaryotic cell lines
[x]	[ ]	Palaeontology and archaeology
[ ]	[x]	Animals and other organisms
[x]	[ ]	Clinical data
[x]	[ ]	Dual use research of concern
[x]	[ ]	Plants

Methods

n/a	Involved in the study	
[x]	[ ]	ChIP-seq
[x]	[ ]	Flow cytometry
[x]	[ ]	MRI-based neuroimaging

Antibodies

Antibodies used	thyroxine (Abcam, ab30833);GFP (Invitrogen, A11122];TFl (Cell Signaling, #12373); pERK (Santa Cruz,sc-377400);Met (Cell Signaling,#8198); phospho-Met (Cell Signaling, #3077 ffor Tyr1234/123S, # 3133 for Tyr 1349); Erk(Cell Signaling ,#9102); phspho-Erk (Cell Signaling ,#9101);Snail (Cell Signaling, #3879,); Cdh1 (Cell Signaling, #3195 ； BD Biosciences,610181） ; β-actin (Cell Signaling, #4967); HSP90(Cell Signaling, #4874); α-Tubulin(Cell Signaling, #2144)

Validation	thyroxine (Abcam, ab30833);https://www.abcam.cn/products/primary-antibodies/thyroxine-antibody-ab30833.html<br>GFP (Invitrogen, A11122];https://www.thermofisher.cn/cn/zh/antibody/product/GFP-Antibody-Polyclonal/A-11122<br>TTF-1(Cell Signaling, #12373); https://www.cellsignal.cn/products/primary-antibodies/thyroid-transcription-factor-1-ttf-1-d2e8-rabbit-mab/12373<br>pERK (Santa Cruz,sc-377400);https://www.scbt.com/p/perk-antibody-b-5?requestFrom=search<br>Met (Cell Signaling,#8198); https://www.cellsignal.cn/products/primary-antibodies/met-d1c2-xp-174-rabbit-mab/8198<br>phospho-Met (Cell Signaling, #3077); https://www.cellsignal.cn/products/primary-antibodies/phospho-met-tyr1234-1235-d26-xp-rabbit-mab/3077<br>phospho-Met (Cell Signaling, # 3133); https://www.cellsignal.cn/products/primary-antibodies/phospho-met-tyr1349-130h2-rabbit-mab/3133<br>Erk(Cell Signaling ,#9102); https://www.cellsignal.cn/products/primary-antibodies/p44-42-mapk-erk1-2-antibody/9102<br>phspho-Erk (Cell Signaling ,#9101);https://www.cellsignal.cn/products/primary-antibodies/phospho-p44-42-mapk-erk1-2-thr202-tyr204-antibody/9101<br>Snail (Cell Signaling, #3879,); https://www.cellsignal.cn/products/primary-antibodies/snail-c15d3-rabbit-mab/3879<br>Cdh1 (Cell Signaling, #3195) https://www.cellsignal.cn/products/primary-antibodies/e-cadherin-24e10-rabbit-mab/3195<br>Cdh1（BD Biosciences,610181） https://www.bdbiosciences.com/zh-cn/products/reagents/microscopy-imaging-reagents/immunofluorescence-reagents/purified-mouse-anti-e-cadherin.610181<br>β-actin (Cell Signaling, #4967); https://www.cellsignal.cn/products/primary-antibodies/b-actin-antibody/4967<br>HSP90(Cell Signaling, #4874); https://www.cellsignal.cn/products/primary-antibodies/hsp90-antibody/4874<br>α-Tubulin(Cell Signaling, #2144) https://www.cellsignal.cn/products/primary-antibodies/a-tubulin-antibody/2144

Eukaryotic cell lines

Policy information about cell lines and Sex and Gender in Research

Cell line source(s)	TPC1 cells were obtained fromthe Chinese Academy of Science in Shanghai ;TOV112D cells were obtained from FuHeng Cell Center.
Authentication	The cell lines were not authenticated.
Mycoplasma contamination	All cell-lines were tested negative for mycoplasma.
Commonly misidentified lines (See ICLAC register)	None of the cell line is listed in ICLAC Register of Misidentified Cell Lines.

Animals and other research organisms

Policy information about studies involving animals; ARRIVE guidelines recommended for reporting animal research, and Sex and Gender in Research

Laboratory animals	Transgenic zebrafish lines tg(tg:GFP),tg(flk1:GFP) and tg(tg:mcherry) aged 24hpf to 1year were used;C57BL/6J aged E11 to 48 week both male and female were used.
Wild animals	No wild animals were used.
Reporting on sex	No sex based analysis were performed.
Field-collected samples	The study did not involve samples collected from the field.
Ethics oversight	All studies were conducted according to the guidelines approved by the ethics committee of Shanghai Ninth People's Hospital.

Note that full information on the approval of the study protocol must also be provided in the manuscript.

Plants

Seed stocks	*Report on the source of all seed stocks or other plant material used. If applicable, state the seed stock centre and catalogue number. If plant specimens were collected from the field, describe the collection location, date and sampling procedures.*
Novel plant genotypes	*Describe the methods by which all novel plant genotypes were produced. This includes those generated by transgenic approaches, gene editing, chemical/radiation-based mutagenesis and hybridization. For transgenic lines, describe the transformation method, the number of independent lines analyzed and the generation upon which experiments were performed. For gene-edited lines, describe the editor used, the endogenous sequence targeted for editing, the targeting guide RNA sequence (if applicable) and how the editor was applied.*
Authentication	*Describe any authentication procedures for each seed stock used or novel genotype generated. Describe any experiments used to assess the effect of a mutation and, where applicable, how potential secondary effects (e.g. second site T-DNA insertions, mosiacism, off-target gene editing) were examined.*
